# Anesthetics inhibit phosphorylation of the ribosomal protein S6 in mouse cultured cortical cells and developing brain

**DOI:** 10.3389/fnagi.2023.1060186

**Published:** 2023-05-16

**Authors:** Matthew B. Friese, Taranjit S. Gujral, Arvind Palanisamy, Brittany Hemmer, Deborah J. Culley, Gregory Crosby

**Affiliations:** ^1^Laboratory for Aging Neuroscience, Department of Anesthesiology, Perioperative and Pain Medicine, Harvard Medical School and Brigham and Women’s Hospital, Boston, MA, United States; ^2^Department of Systems Biology, Harvard Medical School, Boston, MA, United States

**Keywords:** anesthetic neurotoxicity, mTOR, activity dependent cell signaling, developmental neurotoxicity, reverse phase protein array

## Abstract

**Introduction:**

The development and maintenance of neural circuits is highly sensitive to neural activity. General anesthetics have profound effects on neural activity and, as such, there is concern that these agents may alter cellular integrity and interfere with brain wiring, such as when exposure occurs during the vulnerable period of brain development. Under those conditions, exposure to anesthetics in clinical use today causes changes in synaptic strength and number, widespread apoptosis, and long-lasting cognitive impairment in a variety of animal models. Remarkably, most anesthetics produce these effects despite having differing receptor mechanisms of action. We hypothesized that anesthetic agents mediate these effects by inducing a shared signaling pathway.

**Methods:**

We exposed cultured cortical cells to propofol, etomidate, or dexmedetomidine and assessed the protein levels of dozens of signaling molecules and post-translational modifications using reverse phase protein arrays. To probe the role of neural activity, we performed separate control experiments to alter neural activity with non-anesthetics. Having identified anesthetic-induced changes *in vitro*, we investigated expression of the target proteins in the cortex of sevoflurane anesthetized postnatal day 7 mice by Western blotting.

**Results:**

All the anesthetic agents tested *in vitro* reduced phosphorylation of the ribosomal protein S6, an important member of the mTOR signaling pathway. We found a comparable decrease in cortical S6 phosphorylation by Western blotting in sevoflurane anesthetized neonatal mice. Using a systems approach, we determined that propofol, etomidate, dexmedetomidine, and APV/TTX all similarly modulate a signaling module that includes pS6 and other cell mediators of the mTOR-signaling pathway.

**Discussion:**

Reduction in S6 phosphorylation and subsequent suppression of the mTOR pathway may be a common and novel signaling event that mediates the impact of general anesthetics on neural circuit development.

## 1. Introduction

The development and maintenance of neural circuits is highly sensitive to neural activity. General anesthetics, as befits their ability to produce unconsciousness, have profound effects on neural activity. As such, there is concern that these agents, by disrupting neural homeostasis, may alter cellular integrity and interfere with brain wiring, such as when exposure occurs during the vulnerable period of brain development (Perouansky and Hemmings, 2009; Lei et al., 2014). Thus, data from a variety of animal models including rats, mice, *C. elegans*, guinea pigs, pigs, sheep, and non-human primates indicate that anesthetic exposure during early neurodevelopment is associated with widespread apoptosis, changes in dendritic spine density, synaptic strength, and number, and long-lasting cognitive impairment (Rizzi et al., 2008; Liang et al., 2010; Gentry et al., 2013; Olutoye et al., 2015; Whitaker et al., 2016; Coleman et al., 2017; Walters and Paule, 2017). The implications of these data for use of anesthetics in children is a matter of debate (Sun et al., 2016; McCann et al., 2019) but concerns remain that long or multiple general anesthetics may have deleterious long-term consequences (Davidson and Sun, 2018).

One of the most striking and consistent observations of studies thus far is that the deleterious effects of anesthesia on the developing brain do not appear to be limited to specific agents or agents of a particular class. Most general anesthetics work, at least in part, through γ-Aminobutyric acid A (GABA_A_) receptor agonism or NMDA receptor antagonism but agents that act on either or neither of these systems induce neurodevelopmental effects. For instance, agents with strong GABA_A_ receptor agonist properties such as isoflurane, sevoflurane, desflurane, propofol, etomidate, and midazolam cause neuroapoptosis and/or alterations in synapse number, morphology or strength in animals, but so too does ketamine, an agent with only minor effects on GABA_A_ receptors that acts mainly by antagonizing NMDA receptor-mediated neural excitation (Young et al., 2005; Slikker et al., 2007; Brambrink et al., 2010, 2012; Liang et al., 2010; Kodama et al., 2011; Creeley et al., 2013; Zheng et al., 2013; Coleman et al., 2017). While controversial, dexmedetomidine, an α2-adrenergic receptor agonist with little or no effect on either GABA_A_ or NMDA receptors, has also been shown to cause neuroapoptosis in developing neurons (Liu et al., 2016). The fact that these neurodevelopmental effects occur across a broad range of anesthetic/sedative agents with diverse receptor mechanisms of action implies they are mediated by a common, but unidentified, intracellular signaling pathway.

To test that hypothesis, we exposed cortical cells *in vitro* to anesthetics with differing mechanisms of action and quantified signaling pathway responses with a semi-hi-throughput protein quantification assay. The most consistent signaling event was a reduction in phosphorylation of the ribosomal protein S6, which we confirmed also occurs in the brain of neonatal mice anesthetized with sevoflurane. pS6 is a well-known member of the mTOR pathway, and mTOR signaling plays many important roles in brain development and neural homeostasis. Therefore, suppression of this pathway is a potential mechanism for the adverse effects of anesthetics on the developing brain.

## 2. Materials and methods

### 2.1. Mice and anesthesia

The Harvard/BWH Institutional Animal Care and Use Committee approved the protocol and all experiments were conducted according to regulations set forth by the Harvard Medical School and Brigham and Women’s Hospital Standing Committee on Animals.

For *in vitro* experiments, E16 timed pregnant female C57BL/6 mice were purchased from Charles River Laboratory (Wilmington, MA, United States), and cortical cells were isolated as described by others (Kim et al., 2010). Briefly, we grossly dissected the cortex and incubated it for 4 min at 37°C in a solution of 1x Hanks Balanced Salt Solution (HBSS) supplemented with papain (Worthington, NJ, United States) and cysteine (0.3 mg/mL). Protease digestion with papain was terminated with 2x washes in 1x HBSS supplemented with trypsin inhibitor (Sigma, MO, United States) and the cells were then mechanically triturated using a sterile pipette. The cells were counted with a Countess Cell Counter (Invitrogen, CA, United States) and diluted to 1 × 10^6^ cells/mL. 5 × 10^5^ cells were plated in each well of a 24-well culture dish pre-coated with poly-ornithine (Sigma; 30 μg/mL). The cortical cell cultures were maintained in Neurobasal media (ThermoFisher) supplemented with B27 (1:50; ThermoFisher), GlutaMax (1:100; ThermoFisher) and penicillin–streptomycin (1:100; ThermoFisher) and fed with fresh media on the fourth and sixth days *in vitro*.

For *in vivo* experiments, timed pregnant female C57BL/6 mice were purchased from Charles River Laboratory (Wilmington, MA) and housed in individual cages until delivery. A total of 12 neonatal mice (both sexes) remained with their mother until postnatal day 7 (P7) at which time 6 randomly assigned neonatal mice underwent general anesthesia in three independent experiments, with 2 animals undergoing anesthesia and 2 control animals per experiment. The anesthesia exposure consisted of 2.0% sevoflurane in 30% oxygen (2 L/min) for 6 h, mirroring clinically relevant conditions and comparable to other preclinical rodent experiments where developmental neurotoxicity was observed (Zheng et al., 2013). The mice were kept at 37–38°C by placing the anesthetizing chamber in a warm water bath, and skin temperature and sevoflurane concentration were measured at 5-min increments. The remaining six mice served as controls and were treated identically except that they received only 30% oxygen for 6 h. Animals were sacrificed by decapitation at the end of the experiment, with control mice anesthetized briefly prior to sacrifice. We then grossly dissected the cortex and homogenized it at room temperature in a solution containing 1 mL of RPPA buffer [2% sodium dodecyl sulfate (SDS), 50 mM Tris–HCl, 5% glycerol, 5 mM ethylenediaminetetraacetic acid (EDTA), 1 mM sodium fluoride, 10 mM β-glycerol phosphate, 1 mM phenylmethylsulfonyl fluoride (PMSF), 1 mM sodium orthovanadate (Na_3_VO_4_), and 1 mM dithiothreitol (DTT)] and supplemented with protease and phosphatase inhibitor cocktail (ThermoFisher, MA, United States). After homogenization, the samples were centrifuged and the supernatants frozen at −80°C.

### 2.2. Cortical cell anesthesia exposure

Starting on DIV7 and continuing through DIV8, cortical cultures were exposed to propofol, etomidate, or dexmedetomidine. The initial dose in the screening experiments were selected to be supratherapeutic in order to maximize the chance of detecting a signal and then conducted a dose–response study to confirm the results at clinically relevant concentrations of the agents. In brief, neurobasal media was removed and replaced with conditioned media (from sister cultures) that contained one of the following: propofol (2–6 diisopropylphenol; 100 μM; Sigma), etomidate (100 μm; Tocris, MS, United States), or dexmedetomidine (1 μM; Tocris) dissolved to a final concentration of 0.1% dimethyl sulfoxide (DMSO; Sigma). As anesthetics cause profound changes in neural activity, we performed separate experiments to alter neural activity with non-anesthetics. Thus, we treated cells with bicuculline (10 μM; Tocris), a GABA_A_ antagonist that blocks inhibitory signaling and increases neural activity, or a combination of tetrodotoxin (TTX, 1 μM; Tocris), an inhibitor of voltage-gated sodium channels, and 2-amino-5-phosphonopentanoic acid (APV, 100 μM; Tocris), an NMDA receptor antagonist, that completely block action potentials and reduce neural activity. All drugs were dissolved in 0.1% DMSO and cells treated with DMSO alone served as a vehicle control. Cells were treated with each agent for 1, 6, or 24 h, after which they were washed twice with PBS at 4°C, lysed in RPPA buffer, and stored at −80°C. Experimental results are from a total of at least four independent biological replicates (i.e., independent dissections performed on different days). For dose response experiments, cortical cells were treated for 6 h but the dose of etomidate and propofol were varied from 2 to 100 μM (*N* = 4, as indicated), and otherwise treated as above. For all agents, the final concentration of DMSO was 0.1%.

### 2.3. Reverse phase protein arrays

**Figure 1:**
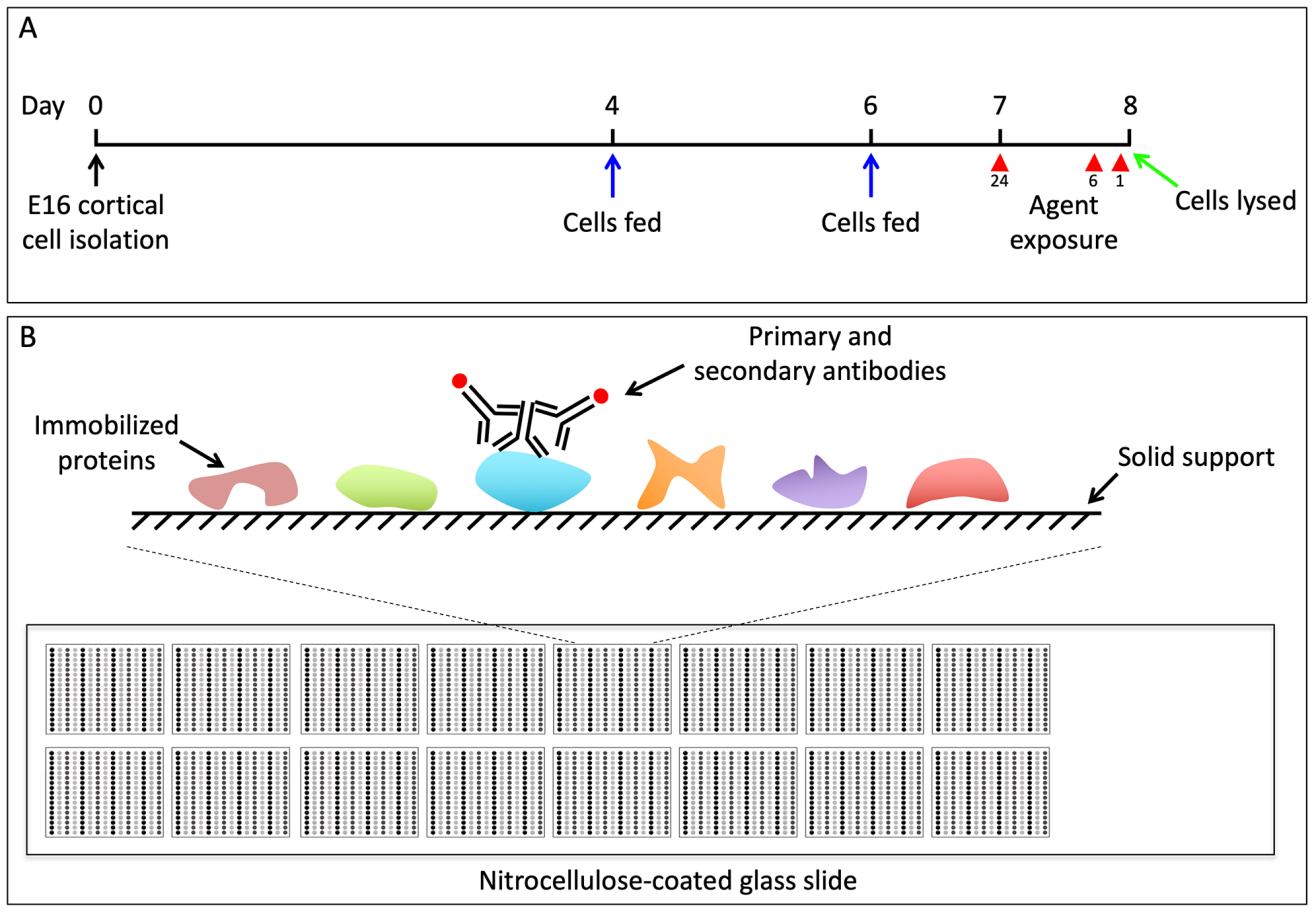
**(A)** Schematic of experimental design. We dissected the brains from pups of timed pregnant C57BL/6 mice at E16.5. The cortical neurons from all pups were pooled and 5 × 10^5^ cells were plated at a density of 1 × 10^6^ cells/mL on 24 well dishes. On DIV4 and DIV6 the media was replenished. On DIV7 we began the 24-h pharmacological exposures, and subsequently began the 6 and 1 h treatments on DIV8. All cells were simultaneously lysed in RPPA buffer on DIV8. **(B)** Outline of RPPA experiments. Each glass microscope slide is embedded with 16 approximately 1 cm × 1 cm nitrocellulose squares. A small amount of protein lysate is dot blotted, or “printed,” onto each of the nitrocellulose squares in replicate, and each nitrocellulose square has the capacity for 225 separate protein lysate dots. Each nitrocellulose square is then blotted with antibodies to the protein of interest, as well as antibodies to actin, which serves as a loading control (Gujral and MacBeath, 2009).

To measure the state of signaling proteins in cultured cortical cells exposed to various IV anesthetics and sedatives, whole cell lysates were subjected to reverse phase protein arrays analysis as described previously (Luckert et al., 2012). Reverse-phase protein arrays are an automated and miniaturized dot-blot assay for protein quantification (Figure 1B). As the system requires only small amounts of protein sample it can be easily scaled up, to test multiple protein samples in a high throughput manner. Briefly, cells were lysed in RPPA buffer. Protein lysates were filter cleared using AcroPrep^™^ Advance 96-Well Filter Plates (Pall Corporation, NY, United States) by centrifuging at 1,962 × g for 4–6 h at room temperature. Total protein amount in lysates was quantified using BCA protein assay kit (ThermoFisher) using manufacturer’s instructions.

Protein microarrays were printed and processed as described in detail previously (Luckert et al., 2012). Protein lysates were printed onto 16-pad nitrocellulose-coated slides (Grace Biolabs, OR, United States) using Aushon 2470 microarrayer (Aushon BioSystems, MA, United States). A total of 6 slides were printed allowing probing with 96 previously validated antibodies (Supplementary Table S1). Slides were stored at −20°C until processing.

### 2.4. Array processing and probing

RPPA slides were washed with 1 M Tris–HCl (pH 9.0) for 2–4 days to remove SDS. Slides were then washed 2–3 times with phosphate-buffered saline (PBS) for 5 min each and blocked with Odyssey Blocking Buffer (OBB, Licor, NE, United States) for 1 h at RT. After blocking, arrays were incubated with primary antibodies in OBB at 4°C overnight. The following day, arrays were washed three times with PBS and incubated with IRDye labeled secondary antibodies in OBB for 1 h at room temperature. Arrays were washed again three times in PBS and once in ddH_2_O and spun dry.

### 2.5. Signal quantification and data analysis

The RPPA slides treated with IR-labeled secondary antibodies were scanned using Licor Odyssey CLX Scanner (LiCOR, NE, United States). Total signal intensity from each spot was quantified using Array-Pro analyzer software package (Media Cybernetics, MD, United States). The measurement of a specific protein from individual sample was then normalized to total β-actin (Sigma).

### 2.6. Western blots

Protein samples were thawed to room temperature and amount of total protein quantified using a BCA protein assay (ThermoFisher). Fifty microgram of total protein was separated by SDS-PAGE (Bio-Rad, CA, United States) and transferred to polyvinylidene difluoride membranes (Bio-Rad) using a semi-dry electrotransfer system (Bio-Rad). The chemiluminescence (ECL) HRP substrate membranes were then blocked in 3% bovine serum albumin (BSA; Sigma) in 0.1% Tween-20 in TBS (TBS-T) for 1 h at room temperature. The blot was then cut into separate pieces based upon the size distribution and the appropriate gel section blotted overnight at 4°C in 3% BSA in TBS-T with the following antibodies: beta-actin (Cell Signaling Technology, MA, United States; Cat #13E5; 1:1,000), pS6 Ser 235/236 (Cell Signaling Technology; Cat #2211; 1:1,000) and cleaved caspase 3 (CC3; Cell Signaling Technology; Cat # 5A1E; 1:200). The following day the blots were washed 3 × 5 min in TBS-T and subsequently blotted with anti-rabbit secondary (Jackson Immuno Research Laboratories, PA, United States; 1:5,000) in 3% BSA in TBS-T for 1 h at room temperature. The blots were washed 3 × 5 min in TBS-T and incubated with SuperSignal West Dura chemiluminescence substrate (ThermoFisher), imaged on ChemiDoc XRS+ imager (Bio-Rad) and analyzed using the Quantity One software package (Bio-Rad).

### 2.7. Data analysis and statistics

Raw data were imported and processed in R (v3.5.1)^1^ or Excel (Mircosoft, WA, United States). In brief, for each pharmacological condition we calculated the log_2_ of the fold-change compared to controls (baseline, unstimulated cortical cells). We then calculated *p*-values using a one-sample *t*-test and subsequently adjusted for the false-discovery rate using the method of Benjamini and Hochberg (1995). Western blot quantification was performed using a 2-sample *t*-test in Excel. Correlation matrix plots were made using the corrplot package for R.

## 3. Results

**Figure 2:**
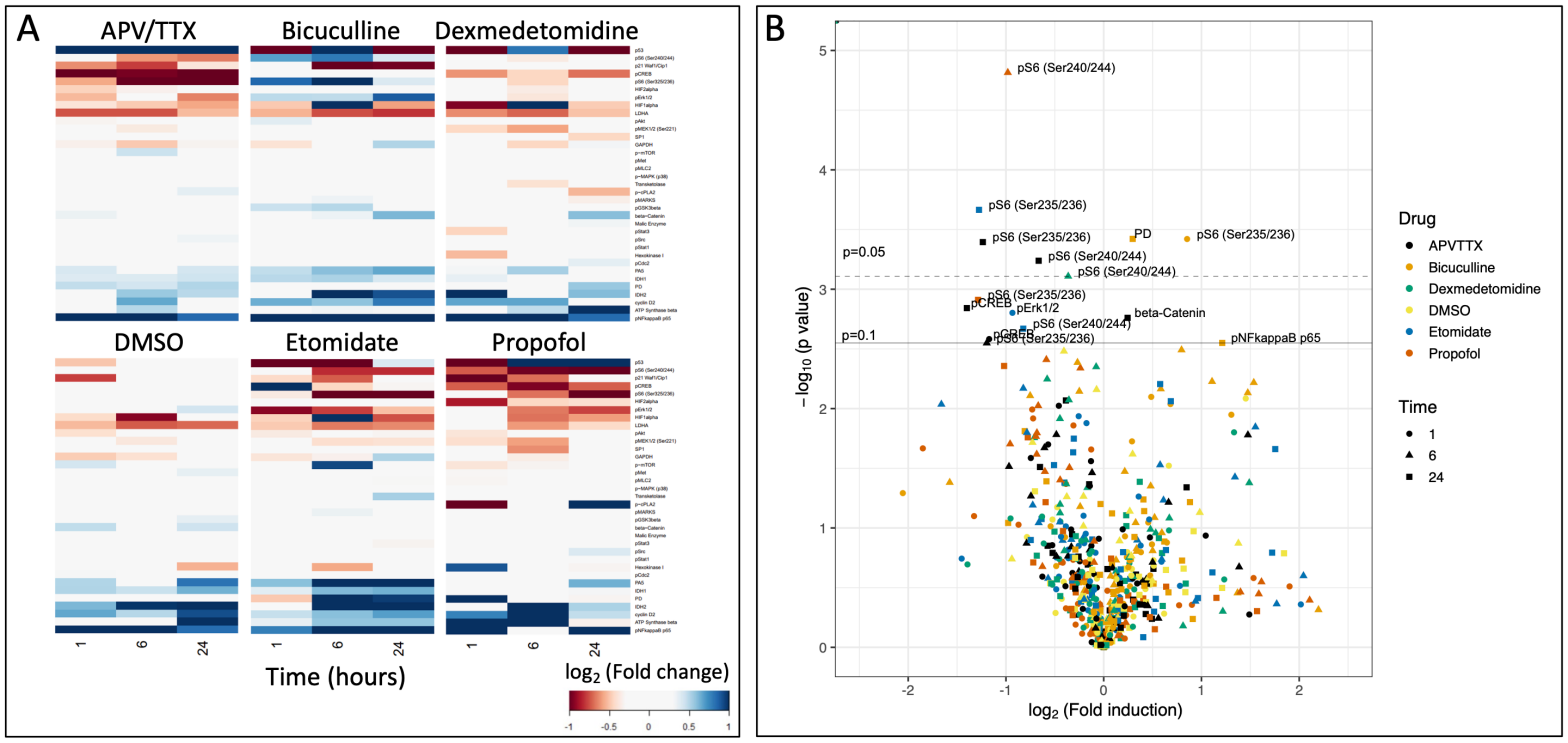
**(A)** Heatmap of raw data from RPPA experiment showing log_2_(fold change) of protein in comparison to baseline after 1, 6, and 24 h of pharmacological exposure. **(B)** Volcano plot of −log_10_(*p-*value) as a function of the log_2_(fold change). Solid line indicates an adjusted *p* = 0.1, and dashed line indicates and adjusted *p* = 0.05 (Benjamini and Hochberg, 1995). There are seven data points that reach the level of significance, six of which are phosphorylation of the ribosomal protein S6.

**Figure 3:**
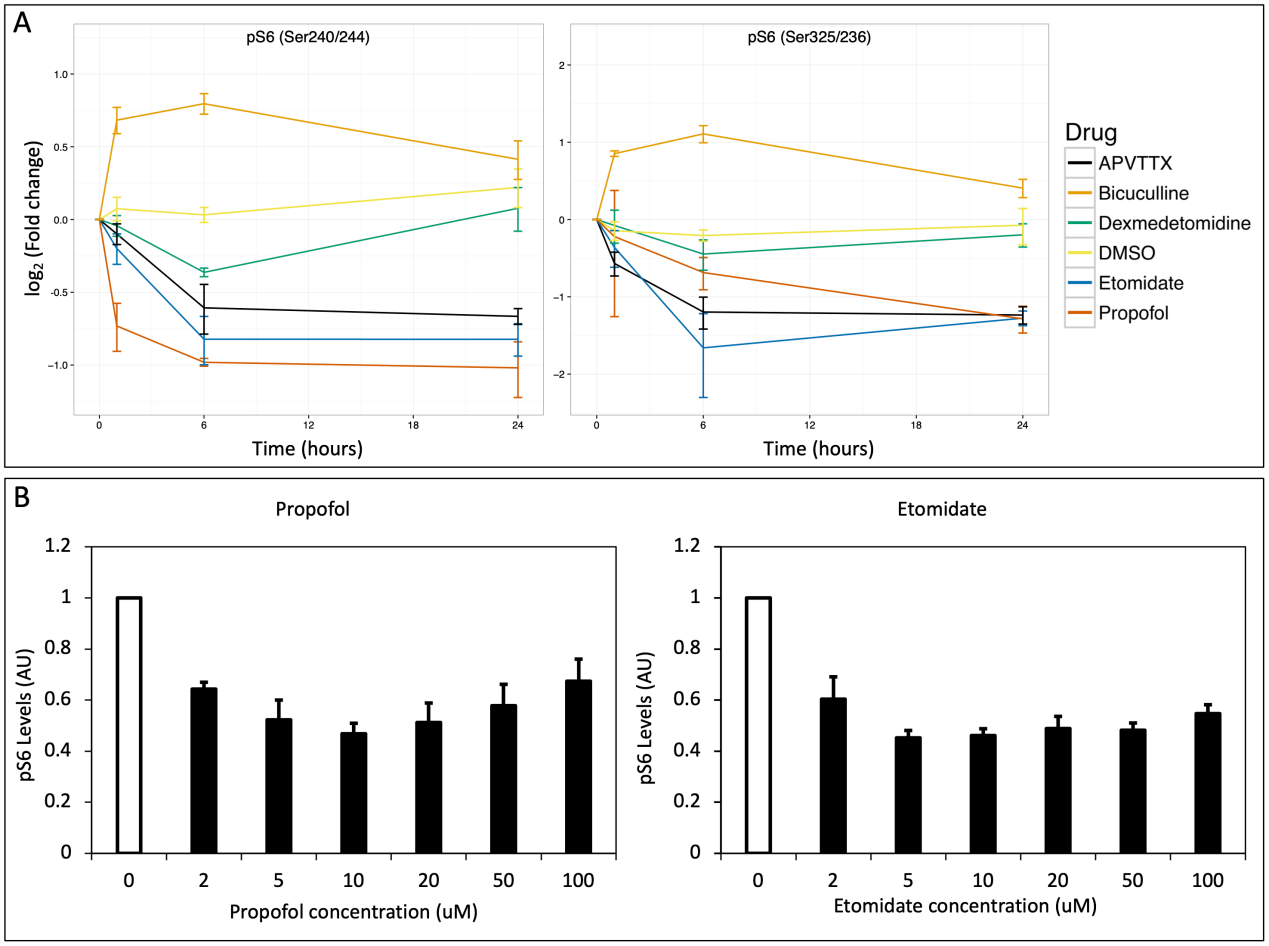
**(A)** Change in pS6 over time. The log_2_(fold change) vs. time is plotted for two antibodies against pS6 that recognize distinct phosphorylation sites (Ser 240/244 and Ser325/326 respectively) and show strikingly similar profiles. Propofol, etomidate, dexmedetomidine and APV/TTX all potently suppress phosphorylation of S6, whereas bicuculline potentiates phosphorylation of S6 and DMSO exhibits no effect on pS6. **(B)** Inhibition of pS6 as a function of concentration for propofol and etomidate. Cortical cells were stimulated for 6 h while varying the concentration of propofol and etomidate from 2 to 100 μM with 0.1% DMSO in neurobasal media. Both propofol and etomidate lead to maximum suppression of pS6 in the range or 2–10 μM. Error bars indicate standard error of mean in both figures.

After correcting for the false discovery rate, we identified signaling events in the *in vitro* experiments that were significantly altered by agent exposure; 6 of these involved changes in phosphorylation of the ribosomal protein S6 (pS6). Propofol and dexmedetomidine reduced pS6 at 6 h (*p* = 0.002 and 0.049, respectively; *N* = 4; Figures 2B, 3A). Likewise, etomidate (*p* = 0.027; *N* = 4) and APV/TTX (noted with two separate antibodies to distinct phosphorylation sites; *p* = 0.033 and 0.040; *N* = 4; Figures 2B, 3A), caused a significant reduction in pS6 at 24 h. In contrast, bicuculline, which increases neural activity (Arnold et al., 2005), increased both pS6 and pyruvate dehydrogenase after 1 and 24 h of treatment (*p* = 0.032 and 0.032, respectively; *N* = 4; Figures 2B, 3A). Given that anesthetics suppress pS6 similar to APV/TTX, whereas bicuculline increases it, these data suggest that anesthetics might be inhibiting pS6 levels by reducing neural activity.

We intentionally used supra-therapeutic anesthetic concentrations in these initial experiments to demonstrate proof-of-concept. Therefore, we next tested a range of clinically relevant concentrations of propofol and etomidate, ranging from 2 to 100 μM propofol or etomidate for 6 h. Propofol produced a significant reduction in pS6 at 2 μM (*p* = 0.005; *N* = 4), 10 μM (*p* = 0.006; *N* = 4), and 20 μM (*p* = 0.047; *N* = 4), and etomidate produced a significant reduction in pS6 at 5 μM (*p* = 0.002; *N* = 4), 10 μM (*p* = 0.002; *N* = 4), 20 μM (*p* = 0.01; *N* = 4), 50 μM (*p* = 0.002; *N* = 4), and 100 μM (*p* = 0.006; *N* = 4) indicating that the decrease in pS6 occurs at brain concentrations of these agents reported to cause clinical effect (Gredell et al., 2004; Benkwitz et al., 2007).

**Figure 4:**
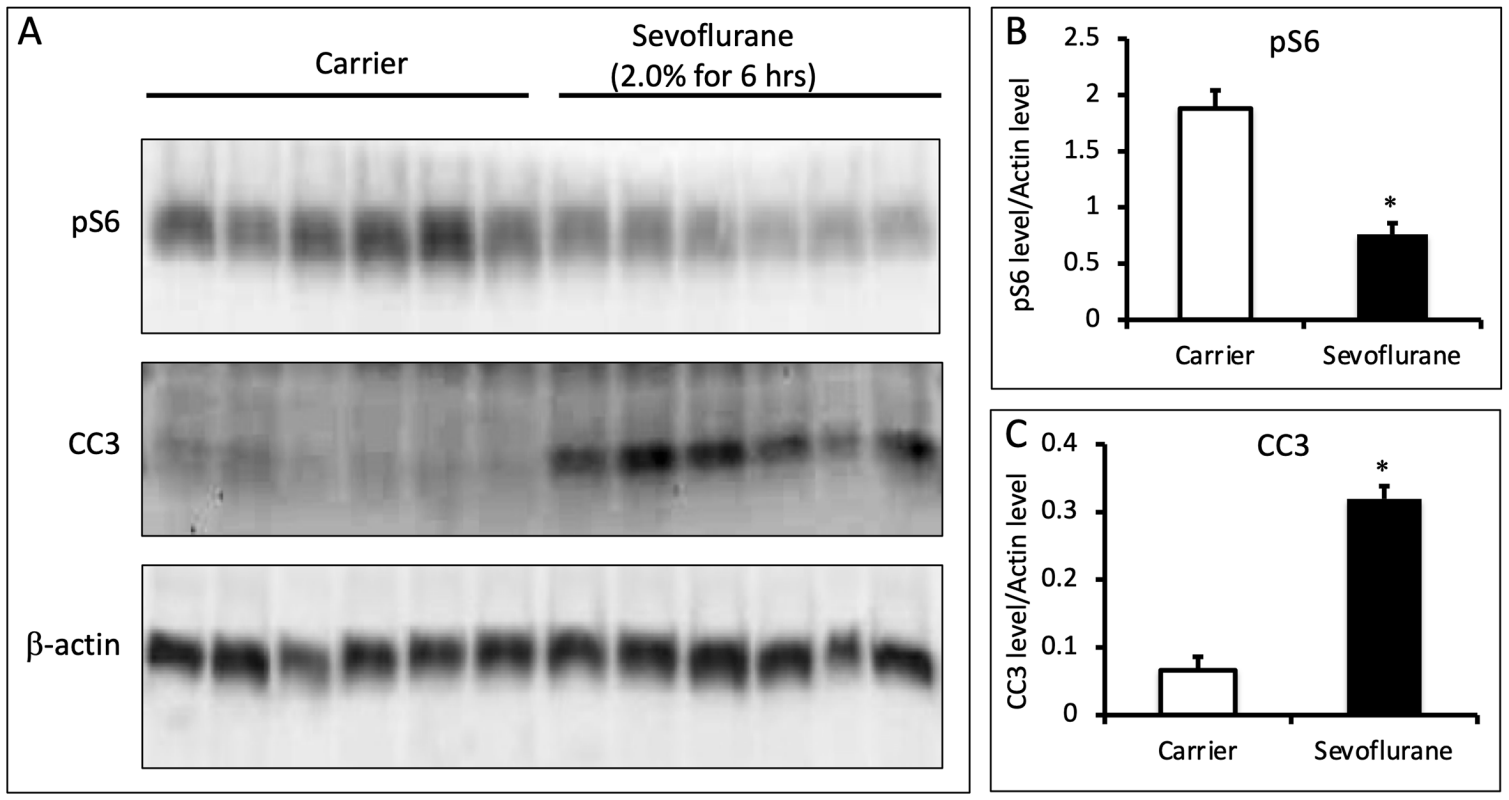
**(A)** Sevoflurane inhibits phosphorylation of S6 in the brains of P7 mice. P7 C57BL/6 mice were anesthetized with 2.0% sevoflurane for 6 h, while control animals were treated identically and received carrier gas but no sevoflurane (*n* = 6 per group). Whole brain lysates were analyzed via quantitative western blots and assayed for the amount of pS6, CC3, and b-actin. **(B,C)** Sevoflurane anesthesia leads to a decrease in pS6 levels and an increase in CC3 (*p* = 0.00015 for pS6 and *p* = 3.2e-6 for CC3; *N* = 6 animals per group; 2 sample *t*-test; error bars indicate SEM; * indicates *p* < 0.05).

To determine if similar changes in pS6 occur *in vivo* during anesthesia, we anesthetized postnatal day 7 (P7) mice with 2% sevoflurane for 6 h and measured pS6 in the brain by Western blot. Sevoflurane is a commonly used volatile anesthetic in children and is reported to produce neuroapoptosis and synaptic abnormalities in neonatal rodents. Compared to control animals that received only oxygen, there was a significant decrease in pS6 in the brain of the sevoflurane anesthetized mice (*p* = 0.00015, *N* = 6 animals per group; Figure 4). Furthermore, there was a significant increase in cleaved caspase 3 in the brain of the anesthetized mice (*p* = 3.21 × 10^−6^, *N* = 6; Figure 4). These data indicate an anesthetic-induced decrease in pS6 occurs in the brain of neonatal animals at a concentration of sevoflurane that also triggers an increase in a marker of apoptosis.

**Figure 5:**
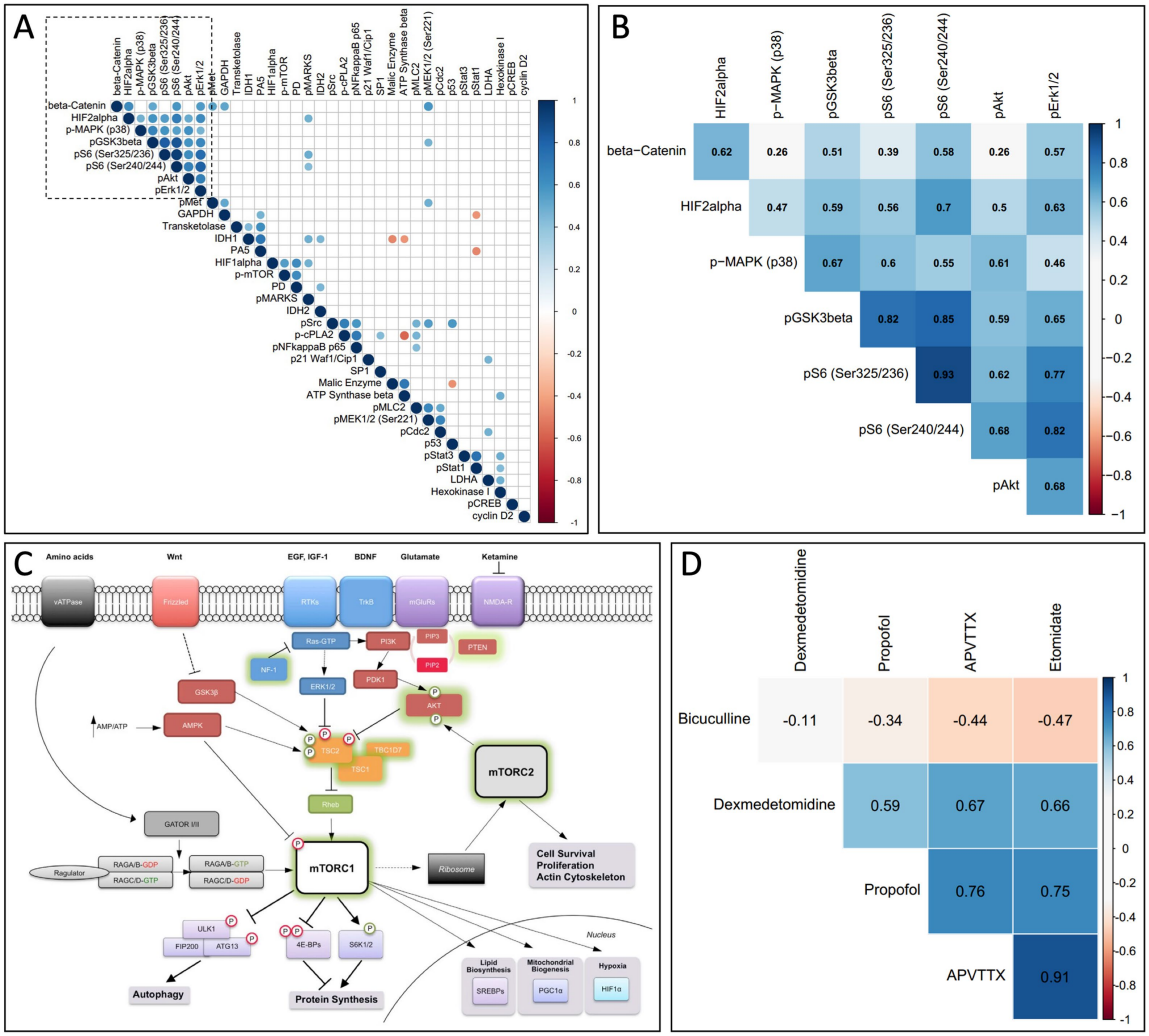
**(A)** Correlation matrix comparing all assays used in RPPA. For each assay on the RPPA matrix we calculated a correlation coefficient for every other assay against all pharmacological conditions and time points, and only significant correlations are shown (*p* < 0.05). The data was clustered using a non-supervised clustering algorithm to identify potential signaling modules. **(B)** A higher magnification of the signaling module outlined by the dashed box in **(A)**, now superimposed with the corresponding correlation coefficient. **(C)** mTOR signaling pathway. A diagram from the mTOR signaling pathway showing that multiple members of the signaling module from **(A,B)** are involved in the mTOR pathway (from Lipton and Sahin, 2014, with permission). **(D)** Correlation matrix comparing the signaling module indicated in **(A,B)** across different pharmacological treatments. Propofol, etomidate, APV/TTX and dexmedetomidine modulate the signaling module from **(A,B)** in a correlated manner. Bicuculline activity on the signaling module indicated in **(A,B)** is anti-correlated with propofol, etomidate and APV/TTX.

Finally, we used a systems-based approach to further evaluate the data from the RPPA experiments. For each signaling event assayed using every time point and pharmacological treatment, we computed a Pearson correlation coefficient for every other assay in a pairwise manner. These correlation coefficients were then clustered using a pairwise clustering algorithm to develop a correlation matrix (Figure 5A), where only statistically significant correlations are shown (*p* < 0.05). This correlation matrix allows us to identify potential signaling modules that may mediate anesthetic-induced signaling. A highly correlated signaling module (identified in Figure 5A and highlighted in Figure 5B) includes pS6 and other cell mediators of the mTOR-signaling pathway (Figure 5C). A correlation matrix comparing how the module highlighted in Figures 5A,B is related across different pharmacological treatments, indicates that propofol, etomidate, dexmedetomidine, and APV/TTX all similarly modulate this pathway (Figure 5D).

## 4. Discussion

Using a hi-throughput protein quantification assay, we demonstrate that clinically relevant concentrations of propofol, etomidate and dexmedetomidine, agents with different mechanisms of action, all suppress S6 phosphorylation in early stage cultured cortical cells. We also extended and validated these results in an vivo model and show that 6 h of sevoflurane anesthesia at clinically relevant concentrations leads to a reduction in pS6 as well as an increase in the apoptosis marker cleaved caspase 3. Phosphorylation of S6 is a well-known component of the mTOR-signaling pathway, and a systems-based approach to analyze the RPPA results further supports our hypothesis by showing that many signaling molecules involved in the mTOR pathway function in a highly correlated signaling module. The activation of this module was correlated between propofol, etomidate, dexmedetomidine and APV/TTX, suggesting that alterations in neural activity level may be responsible for mediating changes in the mTOR pathway. Consistent with this possibility, there was a small but statistically significant increase in expression of pyruvate dehydrogenase, a component of cellular respiration, in cells exposed for 24 h to bicuculline, a drug that increases neural activity. The fact that pyruvate dehydrogenase suppression was not observed with drugs that reduce neural activity suggests a floor in its expression, driven perhaps by ongoing basal, non-activity driven cellular metabolism.

The highly conserved mTOR pathway is involved in many aspects of neural development and homeostasis, making it an intriguing potential target for mediating neurodevelopmental effects of anesthetics (Lipton and Sahin, 2014). Interestingly, the mTOR pathway is implicated in isoflurane and propofol-induced synaptic and behavioral changes, albeit via an aberrant increase in mTOR signaling (Kang et al., 2017; Xu et al., 2018). In that work, the authors exposed older animals (P18) to 4 h of 1.5% isoflurane anesthesia and found 12 days after exposure (P30) that there was an increase in pS6 staining in the dentate gyrus. This increase in pS6 staining was associated with an increase in dendritic length, a reduction in the number of dendritic spines, and later spatial learning defects. Inhibiting the isoflurane-induced activation of the mTOR pathway with rapamycin rescued both the spine loss and behavioral defects, implicating the mTOR pathway in the neurodevelopmental effects of isoflurane, a prototypical volatile anesthetic. Further studies using primary cortical neurons harvested from E18 rats, and exposed to isoflurane between 3 and 7 DIV, showed a similar increase in the number of pS6 positive cells 3+ days after exposure to isoflurane (Kang et al., 2017; Xu et al., 2018).

Our experiments appear to be at odds with the results reported above, inasmuch as we report that propofol, etomidate, and dexmedetomidine reduce pS6 in cultured cells and that sevoflurane produces a similar reduction in the developing brain. However, others have reported that sevoflurane reduces pS6 in the developing brain (Tan et al., 2015; Xu et al., 2018) and that a decrease in mTor signaling, as induced by the mTor inhibitor rapamycin, is associated with apoptosis as identified by markers such as CC3 (Bowling et al., 2018). In addition, there are two important differences between our study and the one mentioned above. First, our anesthesia exposure occurred in DIV8 cortical cells (isolated from E16.5 mice) or P7 animals whereas the prior investigators exposed mice on P18. This is potentially important because the effects of anesthesia on the brain appear to be exquisitely sensitive to developmental age. For instance, apoptosis is first detectable in rodents when anesthesia exposure occurs around birth, reaches a peak when animals are exposed at P7, and then rapidly declines such that it is almost non-existent when exposure occurs on P14 (Yon et al., 2005). Further, others have reported that propofol anesthesia will lead to a loss of dendritic spines when exposure occurs at P5-P10 but that exposure a week later leads to an increase in spines, indicating that in rodents anesthesia has opposite effects depending on developmental age (Briner et al., 2011). Taken together, these data suggest that differences in the mTOR signaling response may underlie some of the age-dependent effects of anesthetic agents on neuronal viability and dendritic spines.

Another potential reason for inhibition of pS6 in our study versus an increase in another may be differences in the post-anesthetic interval, as we assayed pS6 immediately after anesthesia whereas other investigators did so 12 days after exposure in mice, or 3+ days after exposure in rat primary cortical cultures (Kang et al., 2017; Xu et al., 2018). Phosphorylation of S6 corresponds to the activity state of neurons; thus, like other activity-regulated immediate early genes such as Fos, Npas4, and Arc, a change in neuronal action potential firing leads to a corresponding change in phosphorylation of S6 (Kim et al., 2010). Indeed, pS6 is frequently used as an indirect assay for neural activity (Kim et al., 2010; Renier et al., 2016). As such, one would expect phosphorylation of S6 to be low at the end of a prolonged anesthetic, when neural activity levels are generally reduced. Using the same logic, a decrease in pS6 following sevoflurane anesthesia suggests the agent is inhibiting neural activity in the cortex of P7 animals, whereas others have suggested that it does so by increasing excitation (Zhao et al., 2011). On the other hand, increased pS6 days or weeks later might be secondary to, or a marker of, compensatory post-anesthetic neuronal hyperexcitability. Indeed, in the offspring of mice exposed to sevoflurane on post-natal D 16/17, there was an increase in mTor phosphorylation in the brain 24 h later (Ju, 2020). Homeostatic synaptic scaling and several other well-described mechanisms predict that perturbations in neural activity, such as the decrease that typically occurs during general anesthesia, will induce compensatory synaptic changes and a period of neuronal hyperexcitability subsequently (Turrigiano et al., 1998; Turrigiano, 2012; Hengen et al., 2016). Therefore, to the extent pS6 is an activity-regulated immediate early gene, an increase in cortical pS6 days after exposure to general anesthesia with sevoflurane may be a homeostatic response to an earlier period of anesthetic-induced neuronal depression.

Strengths of our study include use of *in vitro* and *in vivo* models, a multiplexed assay system, several anesthetic classes, and non-anesthetic *in vitro* controls. This work also has important limitations. While we identified a common cell-signaling module that is altered similarly across a wide variety of both intravenous and inhalational anesthetics, we have not directly linked this pathway to the central nervous system complications observed after early developmental exposure to these agents. As others have reported neurotoxicity in older animals with an increase in mTOR signaling, the conditions under which an acute change in pS6 is physiologic or detrimental remains to be determined. We did not investigate anesthetic-induced CC3 changes in the *in vitro* experiments because the reagent had not been validated for the RPPA platform, and thus we cannot confirm that propofol, etomidate and dexmedetomidine produced apoptosis-like changes in the *in vitro* model. However, there was an increase in CC3 in the brain of neonatal mice exposed to sevoflurane, suggesting that the pS6 changes are associated with apoptotic events. Finally, this study was largely performed using soluble agents in a tissue culture model. This limits generalizability and requires that results obtained *in vitro* for propofol, etomidate and dexmedetomidine be replicated *in vivo*. Notably, however, we confirmed the results *in vivo* with a widely used inhalational anesthetic (sevoflurane) at a clinically relevant dose.

We conducted these experiments in order to determine whether exposure of cortical cells to anesthetics of different classes alters similar signaling pathways, and thus to provide insight into how agents with different mechanisms of action may mediate similar changes within developing neurons. Here, we find that at clinically relevant concentrations, the intravenous anesthetics propofol, etomidate, and dexmedetomidine suppress pS6 levels in cultured cortical cells, and sevoflurane has a similar effect in the developing mouse brain. The fact that these effects are similar to those seen with the neural activity blockers APV/TTX and opposite those of the neural activity enhancer bicuculline suggest that pS6 is changing in response to alterations in neural activity. Because pS6 is a well-established member of the mTOR signaling pathway, our results suggest that some aspects of anesthetic actions on developing neurons are triggered by reductions in neural activity and subsequent perturbations in this important signaling pathway.

## Data availability statement

The raw data supporting the conclusions of this article will be made available by the authors, without undue reservation.

## Author contributions

MF, TG, DC, and GC conceived and designed the experiments. MF, TG, AP, and BH performed the experiments. MF analyzed the data and drafted the manuscript. All authors revised and approved the final manuscript.

## Funding

This work was funded by the Harvard Anesthesia Training Program (GM007592), a Foundation for Anesthesia Education and Research GEMSSTAR Award, and NIA AG053280 to MF, NIA AG048522 to DC, and NIA AG051812 to GC.

## Conflict of interest

The authors declare that the research was conducted in the absence of any commercial or financial relationships that could be construed as a potential conflict of interest.

## Publisher’s note

All claims expressed in this article are solely those of the authors and do not necessarily represent those of their affiliated organizations, or those of the publisher, the editors and the reviewers. Any product that may be evaluated in this article, or claim that may be made by its manufacturer, is not guaranteed or endorsed by the publisher.

## Footnotes

^1^ cran.r-project.org
