# Supplementary material for: Anesthetics inhibit phosphorylation of the ribosomal protein S6 in mouse cultured cortical cells and developing brain
Source: Front Aging Neurosci. 2023 May 16;15:1060186. doi: 10.3389/fnagi.2023.1060186 (PMC10229047; doi:10.3389/fnagi.2023.1060186)
Supplement: Supplementary file 1 [file Table_1.docx]

## Table 1.

| **Manufacturer** | **Catalog Number** | **Antibody** | **Antibody (Short Name)** |
| --- | --- | --- | --- |
| Cell Signaling Technology | 2211 | Phospho-S6 Ribosomal Protein (Ser235/236) | pS6 (Ser325/236) |
| Cell Signaling Technology | 2101 | Phospho-Src Family (Tyr416) | pSrc |
| Cell Signaling Technology | 4058 | Phospho-Akt (Ser473) (193H12) | pAkt |
| Cell Signaling Technology | 4377 | Phospho-p44/42 MAPK (Erk1/2) (Thr202/Tyr204) (197G2) | pErk1/2 |
| Cell Signaling Technology | 9198 | Phospho-CREB (Ser133) (87G3) | pCREB |
| Cell Signaling Technology | 9582 | beta-Catenin (6B3) Rabbit mAb | beta-Catenin |
| Cell Signaling Technology | 2741 | Phospho-MARCKS (Ser152/156) | pMARKS |
| Cell Signaling Technology | 2831 | Phospho-cPLA2 (Ser505) | p-cPLA2 |
| Cell Signaling Technology | 3033 | Phospho-NF-kappaB p65 (Ser536) (93H1) | pNFkappaB p65 |
| Cell Signaling Technology | 3674 | Phospho-Myosin Light Chain 2 (Thr18/Ser19) | pMLC2 |
| Cell Signaling Technology | 5558 | Phospho-GSK-3beta (Ser9) (D85E12) XP | pGSK3beta |
| Cell Signaling Technology | 9111 | Phospho-cdc2 (Tyr15) | pCdc2 |
| Cell Signaling Technology | 9145 | Phospho-Stat3 (Tyr705) (D3A7) XP | pStat3 |
| Cell Signaling Technology | 9167 | Phospho-Stat1 (Tyr701) (58D6) | pStat1 |
| Cell Signaling Technology | 9215 | Phospho-p38 MAPK (Thr180/Tyr182) (3D7) | p-MAPK (p38) |
| Cell Signaling Technology | 2215 | Phospho-S6 Ribosomal Protein (Ser240/244) | pS6 (Ser240/244) |
| Cell Signaling Technology | 3582 | LDHA (C4B5) Rabbit | LDHA |
| Cell Signaling Technology | 2338 | Phospho-MEK1/2 (Ser221) (166F8) | pMEK1/2 (Ser221) |
| Cell Signaling Technology | 3077 | Phospho-Met (Tyr1234/1235) (D26) XP | pMet |
| Cell Signaling Technology | 5174 | GAPDH (D16H11) XP | GAPDH |
| Cell Signaling Technology | 2024 | Hexokinase I (C35C4) | Hexokinase I |
| Cell Signaling Technology | 2947 | p21 Waf1/Cip1 (12D1) | p21 Waf1/Cip1 |
| Cell Signaling Technology | 2974 | Phospho-mTOR (Ser2481) | p-mTOR |
| Cell Signaling Technology | 3205 | Pyruvate Dehydrogenase (C54G1) | PD |
| Cell Signaling Technology | 8616 | Transketolase | Transketolase |
| Cell Signaling Technology | 12399 | Malic Enzyme 2 | Malic Enzyme 2 |
| Cell Signaling Technology | IDH1 | IDH1 Antibody | IDH1 |
| Cell Signaling Technology | 12652 | IDH2 (D7H6Q) | IDH2 |
| Cell Signaling Technology | HIF1alpha | HIF1alpha | HIF1alpha |
| Cell Signaling Technology | HIF2alpha | HIF2alpha | HIF2alpha |
| Cell Signaling Technology | 9282 | p53 | p53 |
| Cell Signaling Technology | 9389 | SP1 (D4C3) | SP1 |
| Santa Cruz | SC-452 | cyclin D2 Antibody (34B1-3) | cyclin D2 |
| ThermoFisher | MA1-930 | ATP Synthase beta Antibody (4.3E8.D10) | ATP Synthase beta |
| ThermoFisher | PA5-25584 | MARCH5 Polyclonal Antibody | PA5 |
